# Genome-Wide Identification, Expression and Tissue-Specific Epigenetic Modification Analysis of the *Su(var)3-9 SET* Gene Family in Soybean

**DOI:** 10.3390/biology15131085

**Published:** 2026-07-06

**Authors:** Min Wang, Wei Zhou, Zihui Zhang, Lesheng Cao, Lishan Wang, Linan Xie, Junwei Wu, Haoce Xu, Ning Jia

**Affiliations:** 1 College of Agriculture and Forestry Science and Technology, Hebei North University, Zhangjiakou 075000, China; wangmin2451@163.com (M.W.); zhibaowjw@163.com (J.W.); n93664440@163.com (H.X.); 2 College of Pharmacy, Hebei North University, Zhangjiakou 075000, China; 17608203186@163.com; 3 College of Life Science, Northeast Forestry University, Harbin 150040, China; zihuizhang@nefu.edu.cn (Z.Z.); caolesheng@nefu.edu.cn (L.C.); 4 National Key Laboratory of Crop Genetic Improvement, National Engineering Research Center of Rapeseed, Hubei Hongshan Laboratory, Huazhong Agricultural University, Wuhan 430070, China; wanglishan5433@163.com; 5 Institute of Carbon Neutrality, Maoershan National Station for Forest Ecosystem Research, Northeast Forestry University, Harbin 150040, China; linanxie@nefu.edu.cn; 6 Key Laboratory of Sustainable Forest Ecosystem Management-Ministry of Education, School of Ecology, Northeast Forestry University, Harbin 150040, China

**Keywords:** soybean, Su(var)3-9 SET, differential expression, tissue-specific epigenetic modification

## Abstract

Background: *Su(var)3-9 SET* genes encode key histone methyltransferases that catalyze H3K9 methylation, a modification generally associated with heterochromatin formation and transcriptional repression. Methods: We identified 23 *GmSu(var)3-9 SET*s and systematically characterized their tissue-specific epigenetic modifications as well as their phylogenetic relationships, chromosomal distributions, conserved domains, gene structures, GO annotations, collinearity, cis-regulatory elements, and expression profiles across diverse tissues and under salt stress. Results: These genes were divided into seven groups, exhibiting diverse structures and uneven distribution on chromosomes. Gene structure and conserved motif analyses revealed high structural diversity among family members, with variations in intron–exon distribution, conserved motifs, and functional domains. Promoter analysis detected multiple cis elements responsive to light, hormones, and abiotic stresses. Most genes showed preferential expression in meristems, roots, and leaves, and responded to salt stress. Co-expression network analysis revealed that these genes were co-expressed with stress- and development-related genes. *GmSUVH12* histone modifications exhibited obvious tissue specificity. Conclusions: Overall, these results provided insights into the evolutionary and functional roles of *GmSu(var)3-9 SET*s in soybean.

## Simple Summary

*Su(var)3-9 SET* genes encode pivotal histone methyltransferases responsible for H3K9 methylation, which modulates chromatin configuration and gene transcription. Nevertheless, systematic characterization of this gene family remains lacking in soybean. Here, we identified 23 *GmSu(var)3-9 SET* genes and analyzed their phylogeny, gene structure, conserved domains, cis-regulatory elements, expression patterns, and tissue-specific epigenetic modifications. The *GmSu(var)3-9 SET* gene family clustered into seven groups. Most of them exhibited tissue-specific expression. Among them, *GmSUVR5*, *GmSUVH12* and *GmSUVH13* had relatively high expression levels in meristems, roots and leaves, respectively. Further analysis indicated that the tissue-specific expression of *GmSUVH12* might be related to histone modification. The gene expression profiling revealed that these three genes all played an important role in salt stress responses. These findings clarified the evolutionary and functional roles of *GmSu(var)3-9 SET* genes and provided an epigenetic foundation for molecular breeding aimed at improving soybean yield and environmental adaptability.

## 1. Introduction

Histone methylation is catalyzed by histone lysine (K) methyltransferases (HKMTases), a highly conserved family of proteins in eukaryotes that possess histone methylation activity [1]. The catalytic core of HKMTases consists of a conserved ~130 amino acid residue region harboring lysine methyltransferase activity, namely the SET (SUPPRESSOR OF VARIATION, ENHANCER OF ZESTE AND TRITHORAX) domain. The SET domain was first discovered in *Drosophila* at the C-terminal of three regulatory proteins (Su(var)3-9, E(z), and Trithorax) [2,3,4,5]. In *Aradiopsis thaliana* and *maize*, *SET* genes are classified into five categories based on phylogenetic and structural organization, while the *Su(var)* homologous and related genes were clustered into clade 5, all of which possess the PreSET domain (PF05033) [6,7]. More recently, two additional classes (Class VI and Class VII) have been recognized [7,8,9]. In plants, the *Su(var)3-9 SET* gene family can be divided into two major subfamilies, *SUVH* and *SUVR*, based on the presence or absence of the SRA domain (PF02182). The SRA domain directly binds methylated DNA, thereby directing histone methylation in the adjacent chromatin [10], and may contribute to H3K9 methylation-mediated heterochromatin formation [11]. The *Su(var)3-9 SET* gene family in *Arabidopsis thaliana* consists of 15 members, of which 10 are *SUVHs* and five are *SUVRs* [12]. Four AtSUVH proteins have been proven to regulate heterochromatin silencing through their HMTase activity and the regulation of DNA methylation [13,14,15], while SUVR1, 2, and 4 are related to rRNA expression. Experimental verification shows that H3K9me is the preferred substrate of SUVR4, implying that SUVHs and SUVRs may cooperate to achieve different functional H3K9 methylation states [12].

The core of histone modification is in the distinct methylation levels of lysine residues at distinct sites on histone tails. Various lysine methylation marks possess unique functions, and their regulatory effects on gene expression rely on methylation levels (me1, me2, me3) and genomic context. H3K9me2 and H3K9me3 are the markers of constitutive heterochromatin in eukaryotes. H3K27me3 is a repressive histone mark mainly in euchromatic regions, while H3K4me3 and H3K36me3 are activating histone modifications [16]. SUVH4/5/6 proteins recognize methylated cytosines via the SRA domain and catalyze H3K9 dimethylation at adjacent genomic regions, with distinct preferential affinities for different types of DNA methylation (particularly non-CG methylation types) [17,18]. SUVH5 incorporates all the sequences and methylation states, while SUVH4 strongly tends towards CpHpG methylation and SUVH6, on the contrary, strongly prefers both CpHpG and CpHpH methylation [13,19,20]. The N-terminal conserved peptide of SUVH6 is recognized by the BAH (bromo-adjacent homology) domain of ASI1 (ANTI-SILENCING 1), an RNA- and chromatin-binding protein. The interaction in ASI1-BAH and SUVH6, mediated by specific recognition of the arginine residues at the N-terminal of SUVH6, promotes the deposition of H3K9me2 at the target site and influences gene expression in a position-dependent manner [21]. The *suvh4suvh5suvh6* triple mutants exhibited a similar non-CG methylation deficiency phenomenon as the *cmt3* mutant, indicating that SUVH4, SUVH5, and SUVH6 jointly regulate CMT3 activity [22]. SUVH1/3 interacts with SDJ1, SDJ2 and SDJ3 (DNAJ domain-containing homologs) to form the SUVH-SDJ complex, which localizes numerous methylated promoters and possesses transcriptional activation activity. This complex protects methylated promoter genes from transcriptional silencing and plays a crucial role in plant growth and development [23]. The recognition and occupancy of methylated DNA sites by SUVH2 and SUVH9, and the subsequent recruitment of Pol V, are essential for the RdDM pathway. Non-coding RNAs interact with 24-nucleotide small interfering RNAs (siRNAs) bound by ARGONAUTE4 (AGO4) to recruit DRM2, which catalyzes DNA methylation. The resulting methylated DNA further attracts SUVH2 and SUVH9, creating a self-reinforcing loop that promotes the maintenance of DNA methylation at RdDM target loci [24]. SUVH2 has a higher affinity for CG methylation, whereas SUVH9 mainly recognizes SHH methylation [25]. In *Medicago*, MtSUVR2 possesses histone methyltransferase activity and catalyzes the conversion of H3K9me1 to H3K9me2/3 in vitro. Under DNA damage conditions, the proportion of heterochromatin decreases in the *suvr2* mutant, and the level of DSB damage marker γ-H2AX increases, indicating that MtSUVR2 protects DNA from damage through H3K9 methylation [26]. The N-terminal WIYLD domain of SUVR4 binds to ubiquitin; in the presence of free ubiquitin, the product specificity of SUVR4 shifts from dimethylation to trimethylation, converting H3K9me1 into H3K9me3 in vitro [27].

Evidence indicates that AHL10 (AT-Hook Motif Nuclear Localized 10) binds AT-rich DNA sequences located in the matrix attachment regions (MARs) of salt stress-responsive gene promoters and recruits the SUVH2/9 complex. The enrichment of the AHL10–SUVH2/9 complex at these promoter regions promotes H3K9me2 deposition, consequently repressing the transcription of salt stress-responsive genes. However, AHL10 phosphorylation triggers its protein degradation, thereby alleviating the repression of stress-responsive genes and enhancing plant salt tolerance [28]. During *Arabidopsis* embryogenesis, LEC2 (LEAFY COTYLEDON 2) activates the RdDM pathway, leading to the accumulation of high CHH methylation levels. The SUVH-SDJ complex recognizes these hypermethylated regions and recruits AHLs, increasing chromatin accessibility and activating the transcription of totipotent regulatory genes. Concurrently, the complex interacts with LEC2 to form a positive feedback regulatory pathway that further activates totipotent genes and promotes somatic embryogenesis formation [29]. The *Arabidopsis* KYP and SUVH5/6 directly interact with AS1-AS2 (ASYMMETRIC LEAVES1, AS1) and, by altering the histone H3 acetylation and H3K9 dimethylation levels, inhibit KNAT1 and KNAT2 to regulate leaf development [30]. SUVH2 contributes to the full DNA methylation at the AtSN1 (SINE-like retroelement) locus, an endogenous RdDM target site during the early seed development stage, whereas SUVH9 affects RdDM during the nutrient development stage. In the *suvh2* and *suvh9* double mutant, the reduction in RdDM at the AtSN1 site is pronounced [31]. SUVR2 interacts with its paralog SUVR1 to form a protein complex and associates with SNF2-related chromatin remodeling proteins CHR19, CHR27 and CHR28. This complex consequently mediates nucleosome positioning and drives transcriptional silencing [32].

Soybean (*Glycine max* L. Merr) is the primary leguminous crop and a vital global protein source, contributing substantially to human survival and sustainable development [33]. However, the functional characteristics of the *Su(var)3-9 SET* gene family in soybean remain largely uncharacterized. In this study, we identified 23 *GmSu(var)3-9 SET* genes from the soybean genome and analyzed their phylogenetic relationships, chromosomal distributions, gene structures, conserved protein domains, Gene Ontology annotations, tissue-specific epigenetic modification and expression patterns. Collectively, our results lay a foundation for elucidating the biological functions of the *GmSu(var)3-9 SET* gene family in soybean.

## 2. Materials and Methods

### 2.1. Identification of the GmSu(var)3-9 SET Gene Family

The amino acid sequences of the *Su(var)3-9 SET* gene in *Arabidopsis thaliana* and rice were used to query the protein sequences database of the soybean genome with the blastp v2.12.0 program (*p*-value < 1 × 10^−10^) [34,35]. The PreSET (PF05033) and SET domains (PF00856) were used to filter the candidates by HMMER 3.4 (http://www.hmmer.org, accessed on 2 January 2026) [36,37].

The online ExPASy program (https://www.expasy.org/, accessed on 2 January 2026), was used to determine the biochemical characteristics of GmSu(var)3-9 SET proteins, including the number of amino acids, the molecular weight (MW) and predicted isoelectric point (pI) parameters [38].

### 2.2. Phylogenetic Analysis

Phylogenetic relationships among the *GmSu(var)3-9 SET* genes were inferred using the neighbor-joining method with 1000 bootstrap corrections [39]. Full-length amino acid sequences from *Arabidopsis thaliana*, *Glycine max*, and *Oryza sativa* were chosen to construct the phylogenetic tree using MEGA12 software [40].

### 2.3. Gene Structure Analysis

We used the MEME online tool (http://meme-suite.org/, accessed on 5 January 2026) to identify 10 conserved motifs within the *GmSu(var)3-9 SET* gene family in soybean, with an E-value threshold of 10^−5^ [41]. The resulting files were visualized and processed using TBtools-II v2.476 software [42]. Gene structure analysis of the *GmSu(var)3-9 SET* genes was also performed using TBtools-II v2.476 [42], and gene structures were illustrated based on CDS and genomic DNA sequences. Furthermore, the reliability of the predicted protein sequences was verified using the SMART online server (http://smart.embl-heidelberg.de/, accessed on 5 January 2026) [43].

### 2.4. Chromosome Location Analysis, Collinearity Analysis and GO Annotation Analysis

Chromosomal localization data for the *GmSu(var)3-9 SET* genes were retrieved from the JGI Phytozome Ensemble Plants database. Chromosomal distribution maps were constructed using TBtools-II v2.476 software [42]. Full-length amino acid sequences were selected for collinearity analysis with soybean. Collinear relationships were identified using MCScanX and TBtools-II v2.476 software [42,44]. Gene Ontology (GO) analysis of the *GmSu(var)3-9 SET* genes was performed using the SoyBase database (https://www.soybase.org, accessed on 10 January 2026).

### 2.5. Cis-Acting Elements Analysis

We retrieved 2000 bp genomic sequences covering the promoter regions of the *GmSu(var)3-9 SET* gene family in *Glycine max* from the NCBI database. Cis-acting elements were predicted and analyzed using the PlantCARE website (http://bioinformatics.psb.ugent.be/webtools/plantcare/html/, accessed on 10 January 2026) and TBtools-II v2.476 software [42,45].

### 2.6. Expression Pattern Analysis and Co-Expression Network

Transcription data were acquired from the NCBI database (https://www.ncbi.nlm.nih.gov, accessed on 10 January 2026), under accession numbers SRP038111 and PRJNA810576. The expression heatmap was constructed in R, and gene expression levels were quantified based on fragments-per-kilobase-per-million (FPKM) values. The heatmap was generated according to the detected expression profiles. The co-expression network was derived from SoyBase (https://www.soybase.org/, accessed on 10 January 2026), and the method was referred to in Movahedi et al. [46].

### 2.7. Epigenetic Modification Analysis

The soybean reference genome (*Glycine max* Wm82.a2.v1) was used for BSMAP (https://github.com/genome-vendor/bsmap, accessed on 1 March 2026). The genomic DNA was extracted using a CTAB-based method. After Illumina second-generation sequencing was completed, the raw data was obtained. Raw data was subjected to quality control with FastQC 0.12.0 (https://github.com/s-andrews/FastQC, accessed on 1 March 2026); reads were filtered to retain clean reads. The resulting clean reads were then aligned to the soybean reference genome Glycine max Wm82.a2.v1 via Bsmap. Bonferroni correction was applied to adjust *p* values and determine the false discovery rate (FDR). Statistical significance was defined as FDR < 0.05. Perl scripts were employed to calculate methylation levels across CG, CHG and CHH contexts. The analysis methods are based on Zhang et al. [47]. The ChIP procedure is based on Saleh et al. [48]. Chromatin was sheared using a sonicator and incubated together with H3K4me3 (Abcam, ab8580) and H3K27me3 (Abcam, ab6002) to construct a library and then sequenced. The adapters were removed from the raw reads using fastp. Quality control was performed by FastQC. Peak calling was performed using SICER 1.1 (with W = 200, g = 1 window, FDR of H3K4me3 < 0.05; with W = 200, g = 3 windows, H3K27me3, <0.05). The methylation was visualized and analyzed using IGB software 10.2.0 with a wig file. The histone modification was visualized and analyzed using IGV software 2.19.8 with a bam file.

### 2.8. Plant Materials and Salt Stress Treatment

Soybean cultivars Williams 82 (W82) and Jack were used in this study. The experimental method was referenced from Zhang et al. [49]. The plant material was at a temperature of 25 °C under a 13 h light/11 h dark condition. When the soybean had grown to the VE stage, the seedlings were transferred to a hydroponic culture condition with a modified one-half-strength Hoagland nutrient solution. The meristem, leaves, epicotyl, hypocotyl and roots were collected separately in the VC stage to verify the expression of *GmSu(var)3-9 SET* genes in different tissues. When the unifoliate leaves were fully expanded (VC stage), we conducted the salt treatment with concentrations of 160 mM NaCl. When entering the the V2 period under normal conditions, we collected leaves and roots for salt tolerance analysis.

### 2.9. Quantitative RT-PCR (qRT-PCR) for GmSu(var)3-9 SET Genes

Total RNA was extracted from soybean tissues. The SYBR Green I Master mixture (Roche, Basel, Switzerland) was used as the RT-qPCR reagent. All RT-qPCR analyses were carried out using LightCycler 480 SYBR Green I Master (Roche, Basel, Switzerland). The expression levels of specific genes were calculated using the cycle threshold (Ct) 2^−ΔΔCT^ method [50]. The RT-qPCR primers are shown in Table S1. We used the *GmTubulin A* gene as a reference gene [49].

## 3. Results

### 3.1. Identification and Phylogenetic Analysis of the GmSu(var)3-9 SET Gene Family

In order to identify all possible homologs of the *GmSu(var)3-9 SET* gene family in soybean, full-length amino acid sequences of the GmSu(var)3-9 SET proteins determined in *Arabidopsis thaliana* and *Oryza Sativa* were used to query the protein sequence database of the *Glycine max* genome via the blastp program. Sequences harboring both PreSET (PF05033) and SET (PF00856) conserved domains were reserved for subsequent analysis. A total of 23 *GmSu(var)3-9 SET* genes, comprising 15 *GmSUVHs* and eight *GmSUVRs*, were identified and named *GmSUVH1*~*GmSUVH15* and *GmSUVR1*~*GmSUVR8*. The filtered GmSu(var)3-9 SET amino acid sequences were analyzed in the Expasy website to determine isoelectric point (PI) and molecular mass (kDa). The results show that the amino acid length of GmSu(var)3-9 SET proteins ranges from 301 amino acids to 1496 amino acids, and the molecular mass ranges from 33,638.64 to 168,898.58 Da, indicating the substantial variation in the protein properties of GmSu(var)3-9 SET numbers in soybean (Table S2).

**Figure 1:**
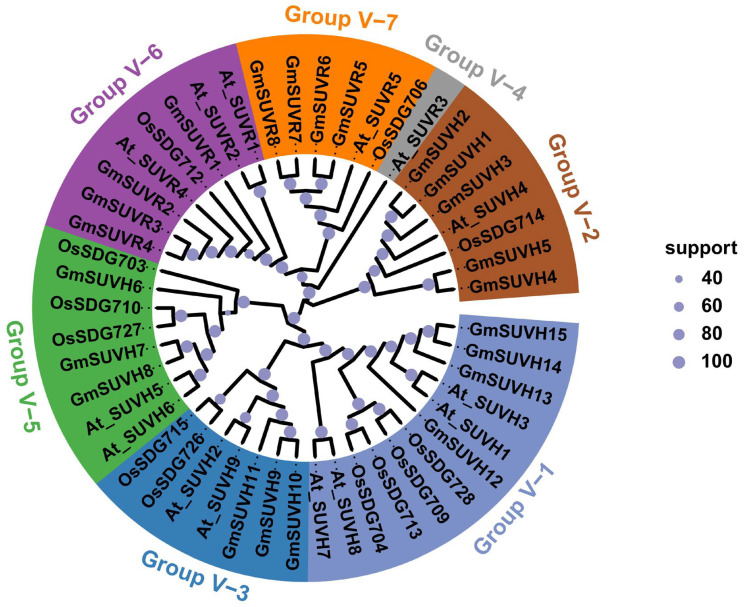
Phylogenetic analysis of Su(var)3-9 SET proteins in *Arabidopsis*, *Oryza sativa* and soybean. The rooted neighbor-joining (NJ) phylogenetic tree of the Su(var)3-9 SET family was clustered with bootstrap values shown for each clade in different sizes. Different groups are marked in different colors.

To dissect the evolutionary history of the Su(var)3-9 SET protein family and to establish phylogenetic relationships, a phylogenetic tree was built in MEGA12 using the neighbor-joining algorithm, with full-length Su(var)3-9 SET protein sequences from soybean, *Arabidopsis* and *Oryza sativa* (Figure 1, Table S3). Based on the branching characteristics and bootstrap values, the *Su(var)3-9 SET* genes are classified into seven major groups. GmSUVH12/13/14/15 belonged to Group V-1, GmSUVH1/2/3/4/5 belonged to Group V-2, GmSUVH9/10/11 belonged to Group V-3, GmSUVH6/7/8 belonged to Group V-5, GmSUVR1/2/3/4 belonged to Group V-6 and GmSUVR5/6/7/8 belonged to Group V-7. *Arabidopsis* SUVH10 was excluded because it might be a pseudogene [51]. Genes within the same group may tend to share similarities in their gene structures, protein architectures, and functional domains.

### 3.2. The Structures of Identified GmSu(var)3-9 SET Genes and Proteins in Soybean

**Figure 2:**
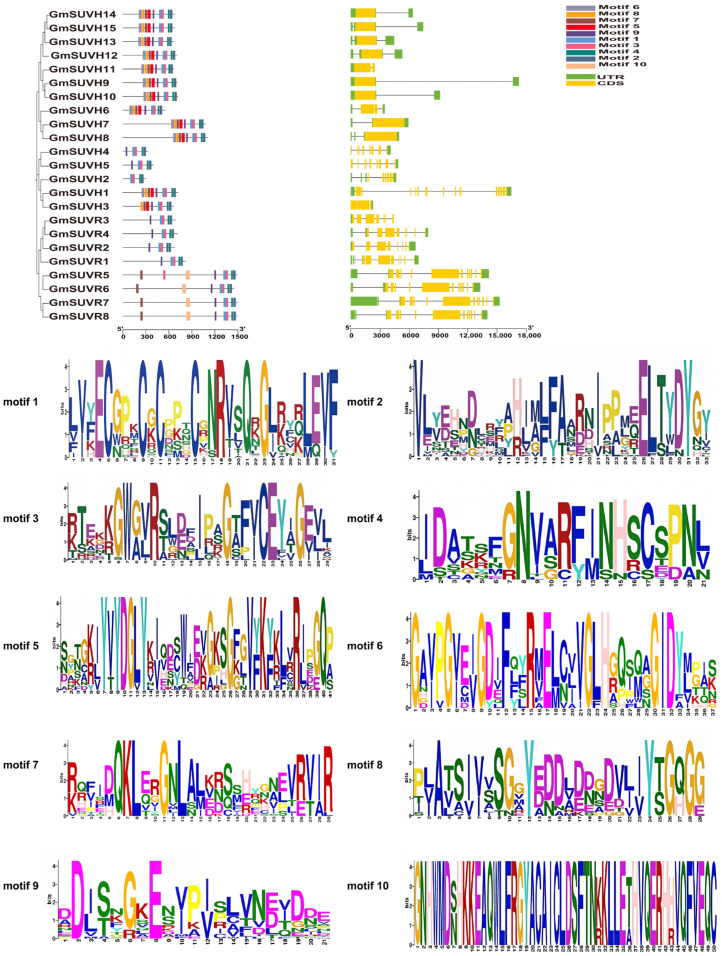
The gene structure and conserved motifs of *GmSu(var)3-9 SET* genes. A total of 10 motifs were identified using the MEME website. The amino acid length was estimated using the scale bar at the bottom. Different colors indicate distinct types of amino acid residues, and the font size reflects the frequency of each amino acid.

Gene structure analysis was performed to characterize the gene length and the exon–intron organization of *GmSu(var)3-9 SET* family members (Figure 2). Gene lengths ranged from 2282 bp to 16,692 bp. In eukaryotes, introns and exons alternate to form genes. Introns exhibit distinct regulatory roles, and their gain or loss may contribute to functional divergence among homologous genes [52]. In plants, up to 60% of genes undergo splicing, with the majority occurring in introns [53]. When intron-mediated enhancement (IME) was employed in *Arabidopsis thaliana*, mRNA levels were markedly increased, accompanied by a 40-fold enhancement of reporter enzyme activity [54]. Most *GmSUVH* members exhibited a simple structure with a few exons, while *GmSUVR* genes contained multiple exons separated by long introns. The *GmSu(var)3-9 SET* gene family in soybean exhibits marked differences in intron and exon numbers from those in *Arabidopsis thaliana*, *Oryza sativa*, and *Populus trichocarpa*, suggesting that the structure of *GmSu(var)3-9 SET* genes is diverse across different species [55].

MEME analysis identified ten conserved motifs (motif1~motif10) in GmSu(var)3-9 SET family proteins (Figure 2). The width, sites, and E-values are provided in Table S4. The conserved motif width ranged from approximately 21 to 50 amino acids, and the number of sites ranged from 4 to 23. Genes clustered closely within the phylogenetic tree shared similar motifs.

**Figure 3:**
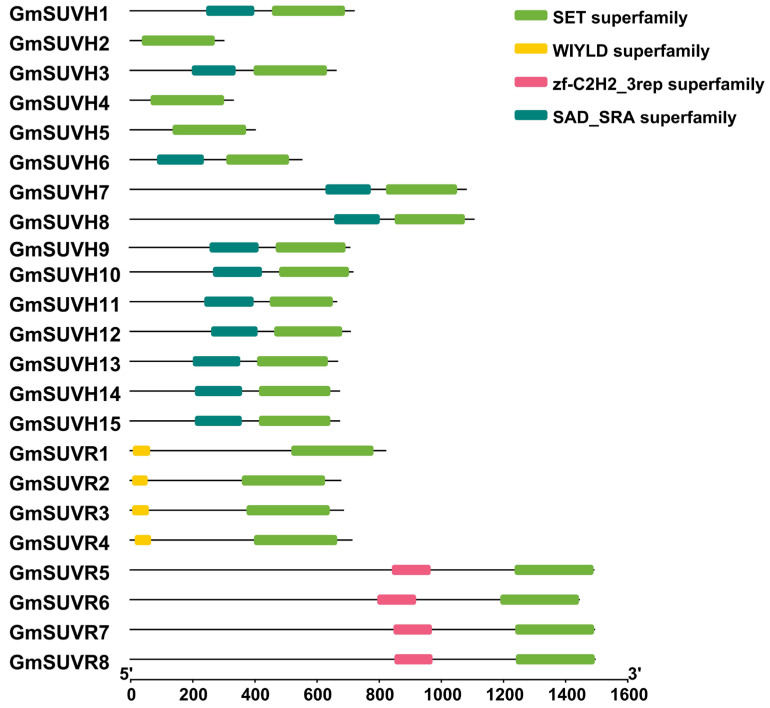
Analysis of the conserved domains in the *GmSu(var)3-9 SET* gene family. All *GmSu(var)3-9 SET* genes contain a SET domain.

To further characterize the structural divergence of the GmSu(var)3-9 SET protein domain, all protein sequences were submitted to the SMART website (Figure 3). All the proteins contained a SET domain. The N-terminal WIYLD domain, which in *Arabidopsis* SUVR4 binds ubiquitin and converts H3K9me1 into H3K9me3 in vitro [27], was present in GmSUVR1/2/3/4. GmSUVR5/6/7/8 have a zf-C2H2 domain at their N-termini. The ZF-C2H2 domain belongs to a type of C2H2 zinc finger (Znf), which facilitates stable binding to target DNA and plays a crucial role in transcriptional regulation [56,57].

### 3.3. Chromosomal Location and Collinearity Analysis of the GmSu(var)3-9 SET Gene Family in Soybean

**Figure 4:**
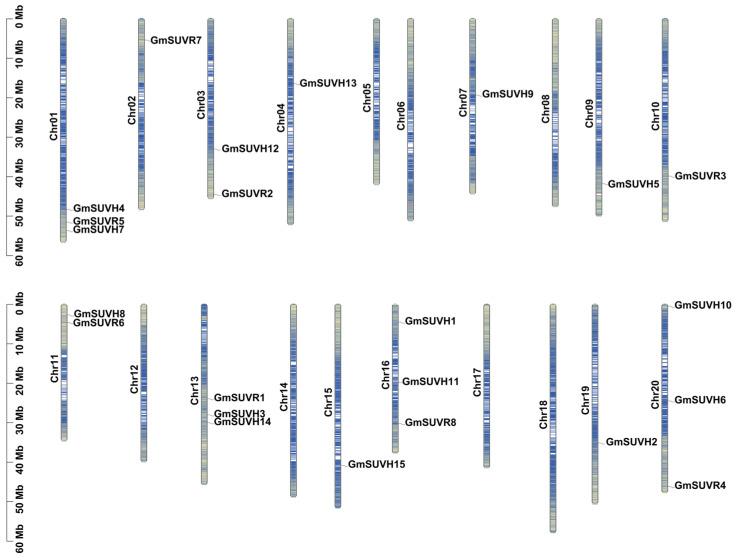
Chromosome location of the *GmSu(var)3-9 SET* gene family in soybean. A total of 23 GmSu(var)3-9 SET genes were distributed on chromosomes 1–20. The chromosome numbers are indicated on the left side. The scale of chromosomal length is shown in Mb.

To investigate the arrangement of 23 *GmSu(var)3-9 SET* genes on chromosomes in the soybean genome, we revealed the chromosomal distribution pattern of this gene family (Figure 4). The gene location information is provided in Table S2. Chromosomes 1, 13, 16 and 20 each contained three *GmSu(var)3-9 SET* genes; chromosomes 3 and 11 each contained two; and chromosomes 2, 4, 7, 9, 10, 15 and 19 each contained one. Gene distribution across chromosomes was independent of chromosomal length. To explore the potential evolution relationship and further compare the *GmSu(var)3-9 SET* gene family collinearity among different species, a comparative analysis was performed between the GmSu(var)3-9 SET protein and homologues from the representative plants, including *Arabidopsis thaliana*, *Oryza sativa*, *Populus trichocarpa*, and *Zea mays* (Figure S1). The results show that there are seven (*Arabidopsis thaliana*), two (*Oryza sativa*), 11 (*Populus trichocarpa*), and three (*Zea mays*) GmSu(var)3-9 SET proteins showing high homology to members from the other four species, respectively. Clear collinear relationships were identified between *GmSu(var)3-9 SET* genes and their orthologs in all examined species, with the most extensive syntenic blocks observed between soybean and *Populus trichocarpa*.

### 3.4. GO Annotations of the GmSu(var)3-9 SET Gene Family

**Figure 5:**
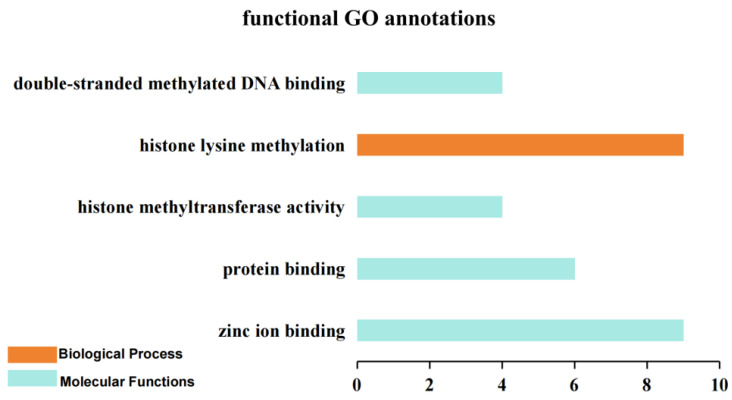
Functional GO annotations of the *GmSu(var)3-9 SET* gene family in soybean. Different biological processes were represented by different colors. The horizontal axis represents the biological process number.

GO enrichment analysis was implemented to predict the potential biological functions of *GmSu(var)3-9 SET* genes (Figure 5, Table S5). *GmSu(var)3-9 SETs* were involved in different biological processes (BP) and molecular functions (MF). All the biological processes were related to histone lysine methylation (GO:0034968). Molecular functions included double-stranded methylated DNA binding (GO:0010385), histone methyltransferase activity (GO:0042054), protein binding (GO:0005515) and zinc ion binding (GO:0008270). The *GmSu(var)3-9 SET* gene family was functionally enriched in epigenetic regulation (histone lysine methylation) and multiple molecular binding/catalytic activities, indicating that GmSu(var)3-9 SET proteins mainly participate in epigenetic regulation that rely on binding and catalytic functions.

### 3.5. The Cis-Acting Regulatory Elements in the Promoter of the GmSu(var)3-9 SET Gene Family in Soybean

**Figure 6:**
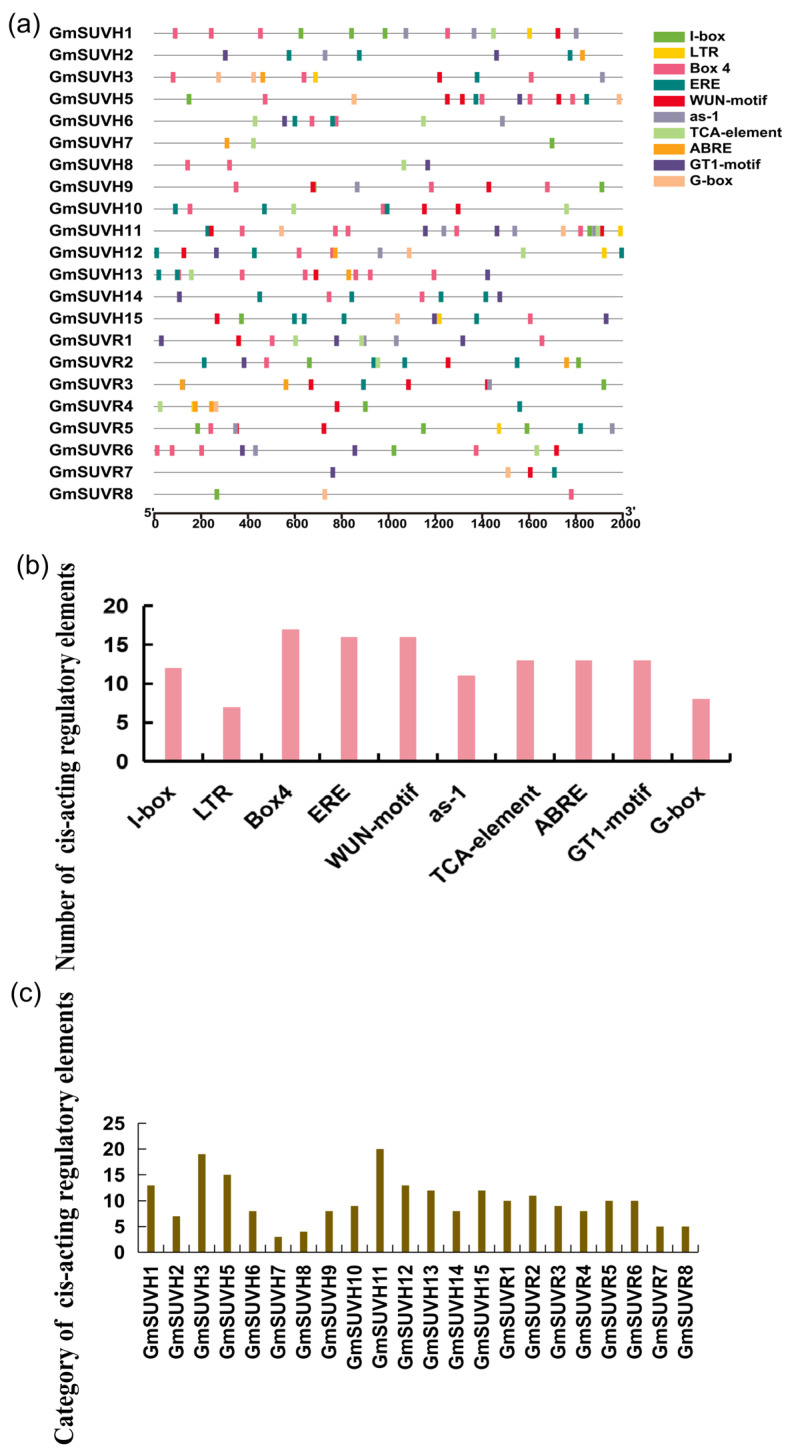
The cis-acting regulatory elements in the promoter of the *GmSu(var)3-9 SET* gene family. Different colors represent different elements. (**a**) Genomic DNA sequences of the 2 kb upstream promoters of the *GmSu(var)3-9 SET* genes to identify cis-acting regulatory elements. (**b**) Number of cis-acting regulatory elements. (**c**) Number of cis-acting regulatory elements present in each *GmSu(var)3-9 SET* gene.

Cis-acting elements in organisms are responsible for binding transcription factors, playing a crucial role in regulating gene expression [58]. To elucidate the regulatory mechanism of the *GmSu(var)3-9 SET* gene family in response to biotic and abiotic stresses, the 2000 bp genomic sequence upstream of *GmSu(var)3-9 SET* promoters was queried using the Plant Care database to identify cis-regulatory elements (CREs). Multiple types of cis-regulatory elements, such as ABRE, ERE, TCA element, I-box, G-box, Box 4, WUN-motif, GT1-motif, as-1, and LTR, were detected within the 2000 bp promoter regions of these genes (Figure 6a). Among family members, *GmSUVH11* carried the greatest abundance of cis-regulatory elements, while *GmSUVH7* harbored far fewer, highlighting heterogeneous transcriptional regulatory complexity across the gene family (Figure 6b,c).

Light-responsive cis-elements, including Box-4, G-box, I-box and GT1-Motif, were detected in the promoter regions of *GmSu(var)3-9 SET* genes. Approximately 74% of these genes contained Box4. Nearly all *GmSu(var)3-9 SET* genes contained at least one light-responsive element in their promoter regions. As a core regulatory component involved in light signaling, the Box4 motif exerts a crucial function in modulating the growth duration of soybean. Loss of this motif leads to a marked upregulation of *GmZTL4* expression and a shortened growth period [59]. Studies have indicated that light-responsive elements (e.g., Box-4 and G-box) are present in the promoter regions of genes involved in drought and salt stress responses, and these elements play critical roles in transcriptional regulation [60,61]. The *MsLEA4-4* promoter contains G-box and abscisic acid response element (ABREs). Overexpression of *MsLEA4-4* in *Arabidopsis* results in more lateral roots, higher chlorophyll content, and significantly higher survival rate under salt stress and abscisic acid treatment conditions relative to the wild type [62]. Under salt treatment, the transcription factors PpWRKY44 and ABRE-BINDING FACTOR3 (PpABF3) promote malic acid accumulation in pear. Specifically, PpABF3 enhances salt-induced malic acid accumulation by targeting the G-box cis-element located in the promoter of *PpWRKY44* [63]. The GT1-motif in the promoter of rice *OsRAV2* directly regulates its salt response [64]. As-1 was found to be linked with oxidative stress responses mediated by salicylic acid [65]. The WUN-motif (wound responsive element) was found in the promoters of 16 *GmSu(var)3-9 SET* genes, implying their potential functional involvement in plant biotic stress responses. LTR was identified as being related to drought induction and the regulation of drought response genes [66]. ABRE, TCA-element and ERE were found to participate in phytohormone regulation. A previous study revealed that the ABRE motif participated in dehydration and salinity stress [67]. ERE and salicylic acid-responsive cis-elements (TCA element) were present in 16 and 13 *GmSu(var)3-9 SET* promoters, respectively. In *Arabidopsis*, *RAP2.11* regulates the expression of *AtHAK5* by binding to the ERE Motif and GCC-box in the *AtHAK5* promoter under low K^+^ conditions and participates in the response to low-potassium conditions [68]. Our results indicate that the *GmSu(var)3-9 SET* gene family may play crucial roles in soybean growth and development, as well as in responses to both biotic and abiotic stresses.

### 3.6. Expression Profiles of the GmSu(var)3-9 SET Gene Family in Soybean

**Figure 7:**
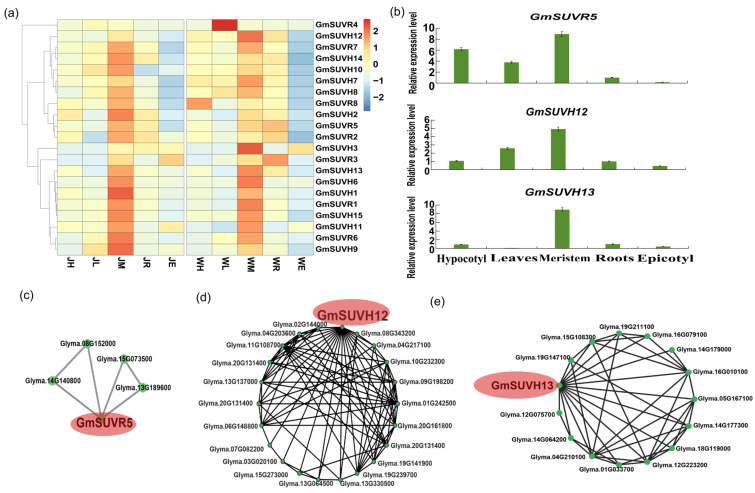
(**a**) The expression levels of GmSu(var)3-9 SET genes in Jack (J) and Williams82 (W). The color gradient from blue to red indicates increasing expression levels. The clustering tree on the left was constructed based on gene expression profiles. The horizontal axis shows the expression of the same gene across various tissues. The column represents the expression of different genes within the same tissue. All the FPKM values were normalized using the Z-score. (**b**) RT-qPCR verification of the expression of *GmSUVR5*, *GmSUVH12* and *GmSUVH13* in leaves, meristem, roots, epicotyl and hypocotyl at the VC stage. M: meristem; U: unifoliate leaves; R: roots; E: epicotyl; H: hypocotyl. Values are means ± SD (*n* = 3). (**c**) Co-expression network for *GmSUVR5.* (**d**) Co-expression network for *GmSUVH12.* (**e**) Co-expression network for *GmSUVH13*.

To better understand the potential roles of the *GmSu(var)3-9 SET* gene family in plant growth and development, expression profiles were examined in different tissues of two representative soybean cultivars, Jack and Williams 82, at the VC stage (Figure 7a, Table S6). The two cultivars were used to assess whether expression profiles differed between varieties. The heatmap showed that *GmSu(var)3-9 SET* gene expression varied substantially across tissues. The gene family was predominantly highly expressed in meristems, with relatively high expression also observed in roots and leaves; these patterns were similar in W82 and Jack. Sixteen and twelve genes showed high expression in Jack and W82 meristems, respectively. *GmSu(var)3-9 SET* genes with high expression levels in meristematic tissues possess promoters enriched in abundant light-responsive cis-acting elements. In meristems, *GmSUVH1* and *GmSUVH11* are both highly expressed, with consistent patterns across varieties. However, the expression of *GmSUVR4* in leaves was notably higher in W82. Although W82 and Jack showed broadly similar *GmSu(var)3-9 SET* gene expression patterns, there are still significant differences between the two varieties. For verification of the RNA-seq data, RT-qPCR was performed on three genes to evaluate the expression pattern of three genes in the roots, leaves, meristem, epicotyl and hypocotyl of W82 (Figure 7b).

Co-expression network analysis based on the global dataset of SoyBase CoNekT was used to screen potential functionally associated upstream and downstream genes that co-expressed with *GmSUVR5*, *GmSUVH12* and *GmSUVH13* (Figure 7c–e, Table S7). The result showed that *GmSUVR5* was co-expressed with Glyma.08G152000, which encodes a calcium-dependent lipid-binding (CALB domain) protein (Figure 7c). A previous study has shown that the knockdown of *HvCaLB1* reduces its salt tolerance [69]. GmSUVH12 was co-expressed with Glyma.04G203600, which encodes an XS zinc finger domain protein (Figure 7d). The AtSGS3 (Suppressor Of Gene Silencing 3) protein belongs to the XS protein family and plays a crucial role in juvenile plant development and trans-acting siRNA production [70]. GmSUVH13 was co-expressed with Glyma.19G147100, a CCCH Zinc-Finger Protein (Figure 7e). CCCH-type zinc finger proteins play an important role in plant development and tolerance to various abiotic stresses, including salt, drought, flooding, low temperature, and oxidative stress [71]. These results demonstrate that the *GmSu(var)3-9 SET* family genes may function in development and stress adaptation through these regulatory partners.

### 3.7. Tissue-Specific Epigenetic Modifications of the Soybean GmSu(var)3-9 SET Gene Family

Plant epigenetics refers to a complex molecular regulatory network that controls gene expression without altering the DNA nucleotide sequence [72]. Key epigenetic regulatory mechanisms in plants include DNA methylation, histone modifications, and non-coding RNAs. Epigenetic modifications play a pivotal role in tissue differentiation and morphogenesis. In *Arabidopsis thaliana*, tissues with different levels of *CLSY* expression exhibited distinct DNA methylation patterns in an RdDM pathway-dependent manner [73]. Nitrogen-induced differentially expressed genes were predominantly enriched in pathways related to nutrient absorption, transport and metabolism in roots, whereas those in shoots were mainly involved in photosynthesis, carbon metabolism and growth and development. This differentiation is determined by organ-specific histone modifications [74].

**Figure 8:**
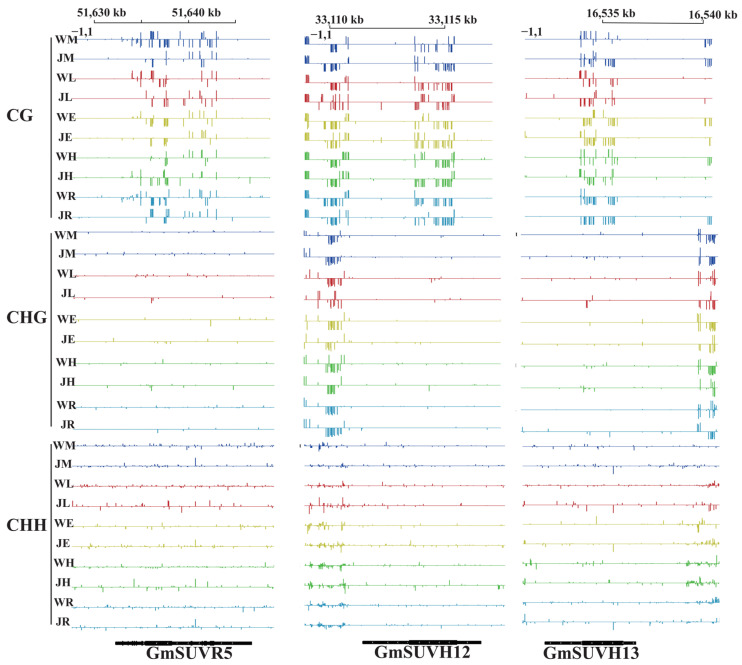
Integrated Genome Browser (IGB) screenshots depicting the CG, CHG, and CHH methylation profiles of the *GmSUVR5*, *GmSUVH12*, and *GmSUVH13* in Jack (J) and Williams82 (W). Different colors represent different tissues. Methylation levels at cytosine sites are shown by vertical bars.

**Figure 9:**
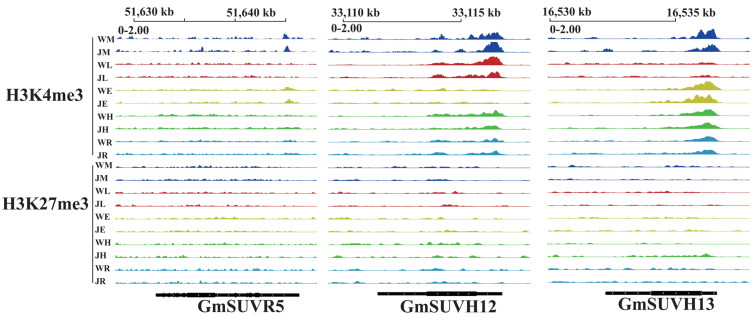
IGV snapshots illustrating the H3K4me3 and H3K27me3 enrichment profiles across the *GmSUVR5*, *GmSUVH12*, and *GmSUVH13* gene loci in Jack (J) and Williams82 (W). Different colors represent different tissues. Histone methylation levels are shown by vertical bars.

In order to better understand whether the expression of the *GmSu(var)3-9 SET* genes was affected by epigenetic modifications, we analyzed the DNA methylation and histone modifications of *GmSUVR5*, *GmSUVH12* and *GmSUVH13* across different tissues and soybean varieties. No significant differences in CG, CHG, or CHH methylation levels were detected among tissues and varieties for *GmSUVR5* (Figure 8, Figure S2). It was plausible that additional regulatory mechanism(s), including repressive TF binding, were also involved in *GmSUVR5* expression. *GmSUVH12* displays elevated CG methylation levels mainly concentrated in the gene body, with no remarkable variation between W82 and Jack (Figure 8, Figure S2), and histone modification analysis further indicated higher H3K4me3 modification levels in leaves and meristem (Figure 9, Figure S3). DNA methylation modification levels of *GmSUVH13* showed no remarkable difference between W82 and Jack (Figure 8, Figure S2). Histone modification analysis indicated higher H3K4me3 modification levels in W82 and Jack (Figure 9, Figure S3). However, *GmSUVH13* had a relatively high expression level only at the meristem, suggesting maybe other mechanisms, including repressive TF binding, are also involved in its low transcript levels.

### 3.8. The Expression of the GmSu(var)3-9 SET Gene Family Under Salt Stress

**Figure 10:**
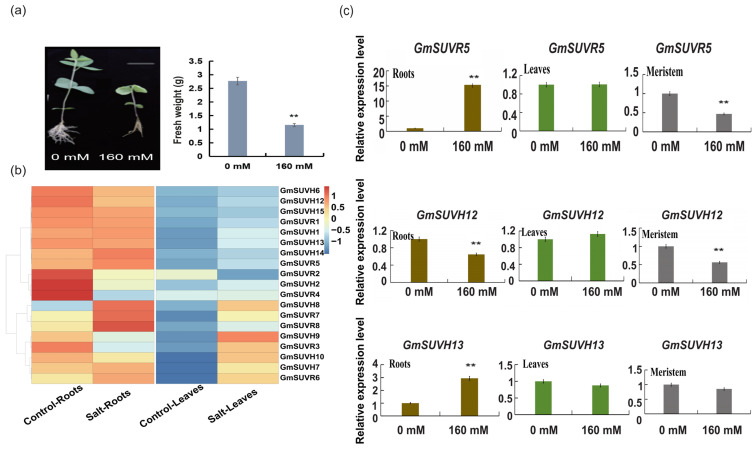
Expression patterns of the *GmSu(var)3-9 SET* genes under salt conditions in Williams82. (**a**) Growth of the Williams82 wild-type plants in liquid medium containing 0 mM NaCl and 160 mM NaCl. (**b**) The expression levels of *GmSu(var)3-9 SET* genes under salt conditions. All the FPKM values were normalized using the Z-score. (**c**) RT-qPCR verification of expression levels of *GmSUVR5*, *GmSUVH12* and *GmSUVH13* in leaves, roots and meristem. Values are means ± SD (*n* = 3). Significant differences are indicated by Student’s *t*-tests (** *p* < 0.01).

Cis-element analysis indicates that the *GmSu(var)3-9 SET* family contains multiple cis-acting elements for abiotic stress responsiveness. To further explore the expression level of this gene family under abiotic stress, we conducted gene expression level analysis and examined the phenotype of soybean under salt stress. A previous study has shown that the protein complex composed of OsBAG4, OsMYB106 and OsSUVH7 regulated the expression of *OsHKT1;5* under salt stress conditions [75]. We first assessed soybean growth under salt stress. Compared with the control group, salt-treated soybeans showed relatively short and visible wilting (Figure 10a). Fresh weight under salt treatment decreased notably (Figure 10a). Transcriptome analysis revealed distinct expression patterns of *GmSu(var)3-9 SET* genes in roots and leaves. Both in control and treatment conditions, a higher number of *GmSu(var)3-9 SET* genes were expressed in roots than in leaves (Figure 10b, Table S6). The relative expression levels of three soybean *GmSu(var)3-9 SET* genes (*GmSUVH12*, *GmSUVH13*, and *GmSUVR5*) were analyzed in roots and leaves under control (0 mM NaCl) and salt stress (160 mM NaCl) conditions (Figure 10c). In roots, salt treatment significantly induced the expression of *GmSUVR5* and *GmSUVH13*. In contrast, the expression of *GmSUVH12* in roots was significantly downregulated under salt stress. In leaves, none of the three genes showed significant changes in response to salt treatment. We also examined the expression of these three genes in meristem. Compared with the control, the expression levels of *GmSUVR5* and *GmSUVH12* were distinctly downregulated under salt treatment. In contrast, the expression of *GmSUVH13* showed only a slight, non-significant reduction under the salt stress condition. These results suggest that the *GmSu(var)3-9 SET* family genes exhibit divergent responses to salt stress.

## 4. Discussion

Histone lysine methylation, as a core component of the epigenetic regulatory network in eukaryotes, plays crucial roles in gene expression regulation, chromatin structure stability, and plant growth, development, and stress adaptation. Its dynamic modification is precisely regulated by histone lysine methyltransferases (HKMTs). The *Su(var)3-9 SET* gene family, an important branch of HKMTs, participates in biological processes such as heterochromatin silencing and gene transcription regulation through SET domain-mediated histone methylation activity [76]. The soybean genome contained 23 *GmSu(var)3-9 SET* family members, including 15 *GmSUVH* and eight *GmSUVR* genes — a number notably higher than that in *Arabidopsis* (15) and rice (12). The collinearity analysis results showed that the genes of the *GmSu(var)3-9 SET* gene family in soybean have a relatively higher degree of collinearity with the *Su(var)3-9 SET* gene family genes in *Populus trichocarpa*. These differences may be closely related to the two whole-genome duplication events that soybean has experienced during its evolution. Zhu et al. [55] analyzed the homologous sequences of Su(var)3-9 SET proteins in representative terrestrial plants and classified Su(var)3-9 SET into seven subtypes. Except for Group V-4, the remaining subtypes are referred to as cSUVH (core Su(var)3-9 homologues and related genes). Phylogenetic analysis in this study indicated that the soybean *Su(var)3-9 SET* gene family had a relatively close genetic relationship with the homologous genes of *Arabidopsis thaliana*. Apart from the absence of Group V-4, all subtypes were represented. GO enrichment analysis revealed that *GmSu(var)3-9 SET* genes mainly participate in biological processes such as histone lysine methylation (GO:0034968), methylated DNA binding (GO:0010385), etc., and had histone methyltransferase activity (GO:0042054) and zinc ion binding activity (GO:0008270). The functional annotations of this gene family were highly consistent with those of the homologous genes in *Arabidopsis thaliana* and rice, indicating that the core functions of the *Su(var)3-9 SET* gene family were evolutionarily conserved. In summary, the *GmSu(var)3-9 SET* gene family had preferentially retained its core functions during the genome duplication event of soybeans to ensure regulatory pathway stability, while also undergoing diversification through genomic rearrangement and gene replication differentiation.

Tissue expression profiling indicated that the *GmSu(var)3-9 SET* genes were highly expressed in the meristems, roots, and leaves, with similar expression patterns across different varieties (Jack and Williams 82), suggesting conserved regulatory function during the growth and development. Meristematic tissue, as a crucial site for cell division and differentiation, exhibits more active chromatin dynamics and gene expression regulation. The high expression of the *GmSu(var)3-9 SET* genes in this tissue may affect cell differentiation and organ formation by regulating the histone methylation status. This observation was consistent with the findings in *Arabidopsis*, in which *Su(var)3-9 SET* genes were involved in somatic embryogenesis and leaf development [29,30]. In *Arabidopsis thaliana*, AHL10 promotes the modification of H3K9me2 in the promoter region of salt-responsive genes by recruiting the SUVH2/9 complex, thereby inhibiting gene expression. Under salt stress, AHL10 is degraded, relieving this repression and enhancing salt tolerance [28]. The GmSu(var)3-9 SET proteins in soybean may regulate the expression of salt-responsive genes through a similar mechanism: under salt stress, some members of GmSUVH show decreased expression, resulting in a lower level of H3K9me2 modification, activating the transcription of downstream salt-tolerant genes, thereby enhancing the adaptability of soybeans to salt stress. Additionally, the N-terminal WIYLD domain of *Arabidopsis* SUVR4 may regulate H3K9 methylation levels through ubiquitin binding [27]. Furthermore, the promoter regions of highly expressed genes were enriched in light-responsive cis-elements (such as Box4 and G-box), and Box4 had been proven to be involved in the regulation of soybean growth stages [59], suggesting that *GmSu(var)3-9 SET* genes might regulate soybean growth and development in response to light signals. *GmSUVR5*, *GmSUVH12*, and *GmSUVH13* exhibited potential functional associations with calcium-binding, XS-domain, and CCCH zinc-finger proteins via co-expression, implying that the *GmSu(var)3-9 SET* family might integrate epigenetic regulation with calcium signaling, small RNA biogenesis, and abiotic stress responses.

Plant epigenetics, which includes DNA methylation, histone modifications and non-coding RNAs, serves as a critical regulator of gene expression. In *Arabidopsis* root meristem cells, the columella root cap exhibited substantial hypermethylation accompanied by small RNA enrichment, alongside distinct tissue-specific cell identities [77]. Our analysis of three representative *GmSu(var)3-9 SET* genes revealed minimal variation in DNA methylation levels across tissues and soybean varieties, whereas histone modifications displayed distinct tissue-specific patterns. These results implied that histone modification, rather than DNA methylation, might act as the primary epigenetic determinant underlying the tissue-specific regulation of *GmSu(var)3-9 SET* genes in soybean.

In conclusion, this study provided an important reference for a deeper understanding of the functions and evolution of the *GmSu(var)3-9 SET* gene family in soybean. The tissue-specific expression patterns and epigenetic modification characteristics of these genes offered candidate genes and a theoretical basis for subsequent functional verification and molecular breeding. Further analysis of the biological functions of the members of this family, new ideas and technical support for soybean stress-resistant breeding can be expected.

## 5. Conclusions

Twenty-three *Su(var)3-9 SET* genes were identified in the soybean genome, including 15 *SUVH* genes and eight *SUVR* genes. Analyses of gene structure, conserved motifs, and domains have shown that members of the *GmSu(var)3-9 SET* gene family were relatively evolutionarily conserved. All identified *GmSu(var)3-9 SET* genes contained a SET domain, with some members also possessing either a WIYLD domain or a zf-C2H2 domain. Expression patterns of these genes exhibited tissue specificity. Tissue-specific epigenetic modifications, particularly histone modifications, displayed distinct patterns across soybean tissues. In summary, these findings indicated the potential roles of the *GmSu(var)3-9 SET* gene family in soybean growth, development, and stress responses.

## Data Availability

The original contributions presented in this study are included in the article and Supplementary Materials. Further inquiries can be directed to the corresponding author.

## Supplementary Materials

The following supporting information can be downloaded at: https://www.mdpi.com/article/10.3390/biology15131085/s1, Table S1: The primers of qRT-PCR; Table S2: *GmSu(var)3-9 SET* gene family screening, naming and location; Table S3: Su(var)3-9 SET GeneBank numbers of *Arabidopsis thaliana* and *Oryza sativa*; Table S4: E-values, sites and width of conserved motifs in *GmSu(var)3-9 SET* genes; Table S5: The functional annotations of the *GmSu(var)3-9 SET* genes in soybean; Table S6: FPKM of the *GmSu(var)3-9 SET* genes in soybean; Table S7: Genes present in co-expression network; Figure S1: Collinearity analysis of the *GmSu(var)3-9 SET* gene family between *Glycine max* and four representative plant species (*Arabidopsis thaliana*, *Oryza sativa*, *Populus trichocarpa*, and *Zea mays*); Figure S2: Statistics of CG, CHG and CHH methylation levels of *GmSUVR5*, *GmSUVH12* and *GmSUVH13* in Jack (J) and Williams82 (W); Figure S3: Statistics of H3K4me3 methylation levels of *GmSUVH12* and *GmSUVH13* in Jack (J) and Williams82 (W).
