# Supplementary material for: Genome-Wide Identification, Expression and Tissue-Specific Epigenetic Modification Analysis of the Su(var)3-9 SET Gene Family in Soybean
Source: Biology (Basel). 2026 Jul 6;15(13):1085. doi: 10.3390/biology15131085 (PMC13360523; doi:10.3390/biology15131085)
Supplement: Supplementary file 1 [file biology-15-01085-s001.zip › Figure S1.pdf]

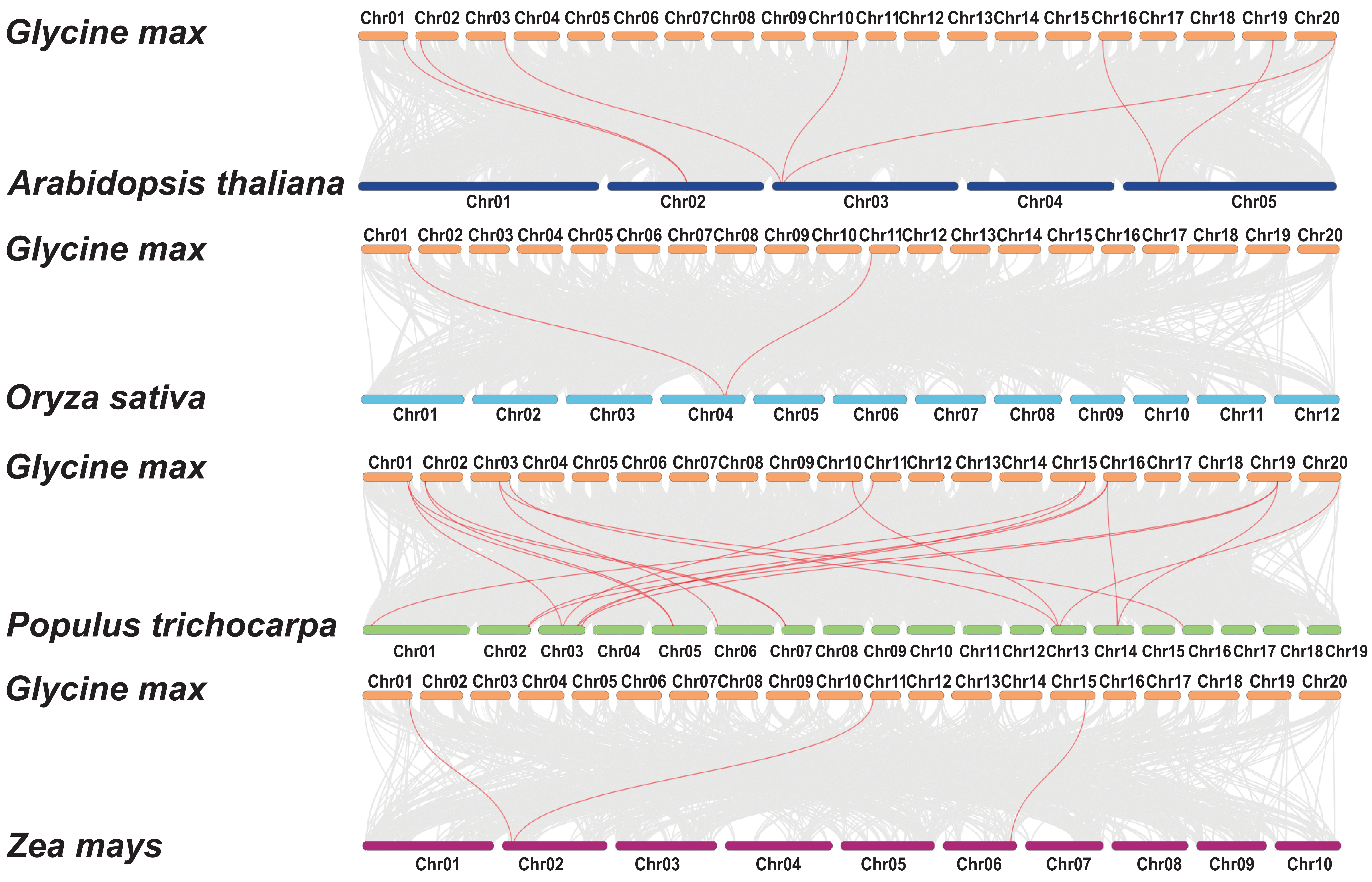

Figure S1. Collinearity analysis of the *GmSu(var)3-9 SET* gene family between *Glycine max* and four representative plant species (*Arabidopsis thaliana*, *Oryza sativa*, *Populus trichocarpa*, and *Zea mays*).
