# Supplementary material for: Genome-Wide Identification, Expression and Tissue-Specific Epigenetic Modification Analysis of the Su(var)3-9 SET Gene Family in Soybean
Source: Biology (Basel). 2026 Jul 6;15(13):1085. doi: 10.3390/biology15131085 (PMC13360523; doi:10.3390/biology15131085)
Supplement: Supplementary file 1 [file biology-15-01085-s001.zip › Figure S2.pdf]

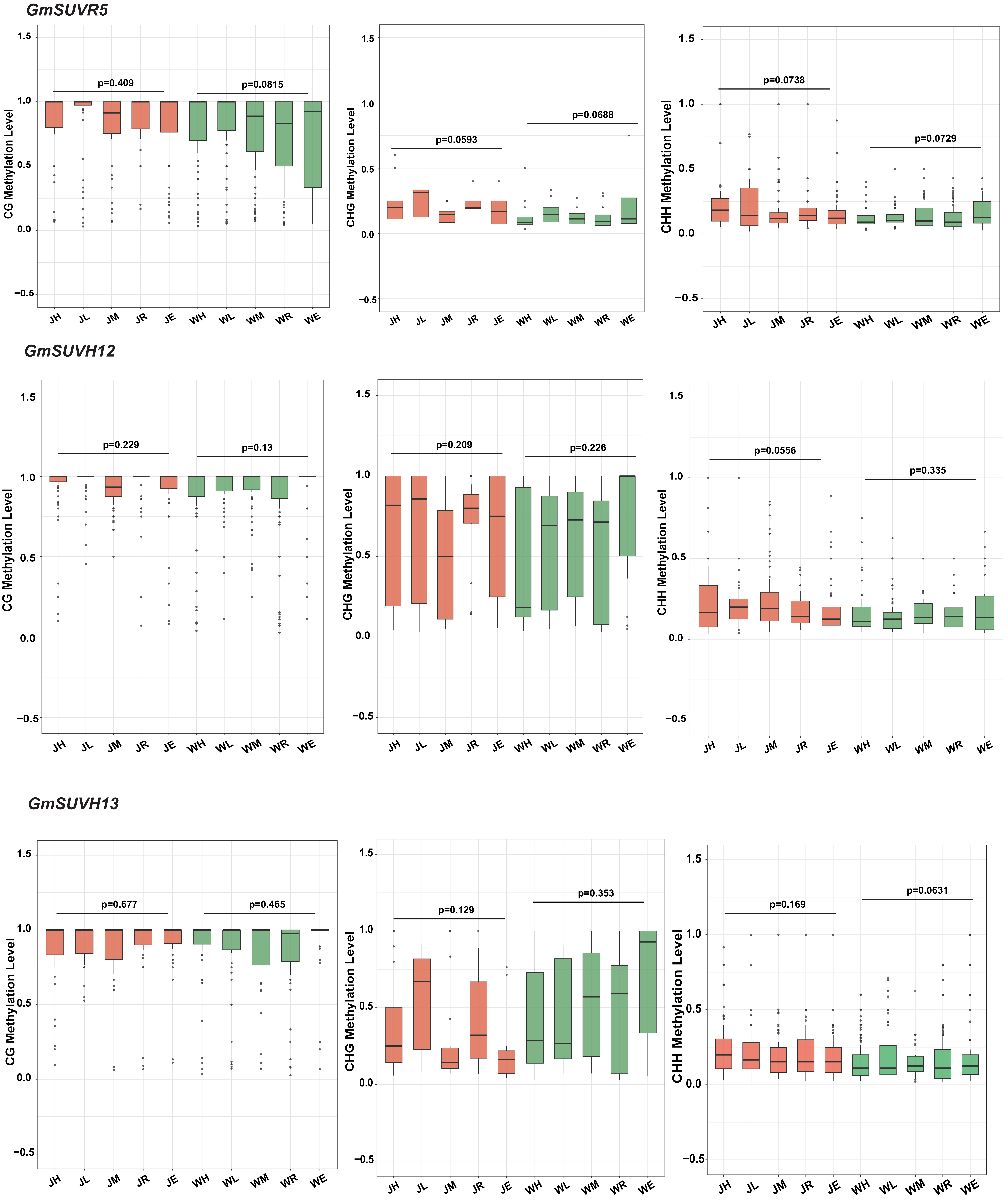

Figure S2. Statistics of CG, CHG and CHH methylated levels of *GmSUVR5*, *GmSUVH12* and *GmSUVH13* in Jack (J) and Williams82 (W). The ANOVA analysis revealed that the differences in the levels of CG, CHG, and CHH methylation among the various tissues and varieties for *GmSUVR5, GmSUVH12, and GmSUVH13* were not significant.
