# Supplementary material for: Genome-Wide Identification, Expression and Tissue-Specific Epigenetic Modification Analysis of the Su(var)3-9 SET Gene Family in Soybean
Source: Biology (Basel). 2026 Jul 6;15(13):1085. doi: 10.3390/biology15131085 (PMC13360523; doi:10.3390/biology15131085)
Supplement: Supplementary file 1 [file biology-15-01085-s001.zip › Figure S3.pdf]

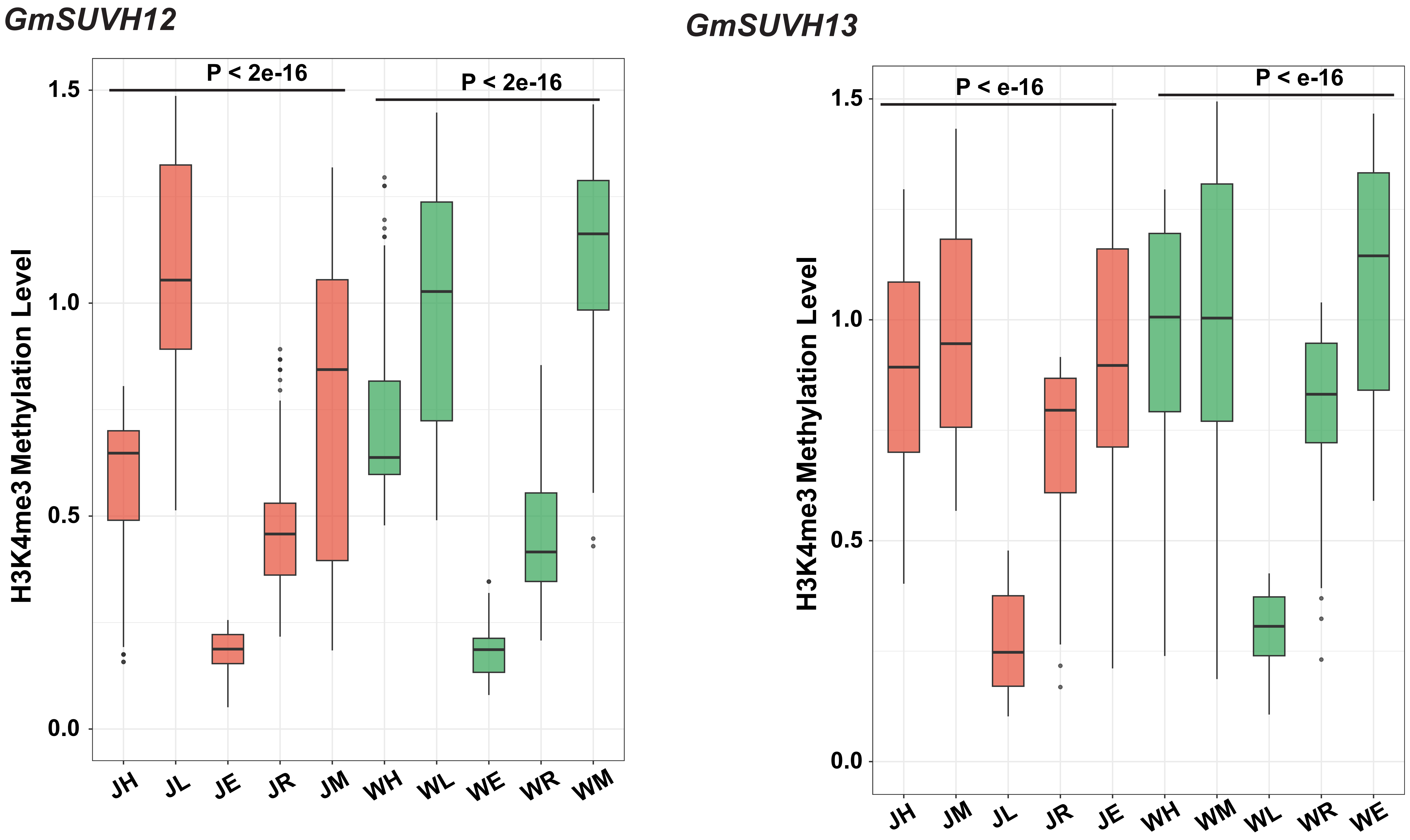

Figure S3. Statistics of H3K4me3 methylation levels of *GmSUVH12* and *GmSUVH13* in Jack (J) and Williams82 (W). The ANOVA analysis revealed that the differences in the levels of H3K4me3 methylation among the various tissues and varieties for *GmSUVH12* and *GmSUVH13* were significant.
